# Vaccinia virus virulence factor N1 can be ubiquitylated on multiple lysine residues

**DOI:** 10.1099/vir.0.065664-0

**Published:** 2014-09

**Authors:** Carlos Maluquer de Motes, Torben Schiffner, Rebecca P. Sumner, Geoffrey L. Smith

**Affiliations:** 1 Department of Pathology, University of Cambridge, Tennis Court Road, Cambridge CB2 1QP, UK; 2 Department of Virology, Imperial College London, St Mary’s Campus, Norfolk Place, London W2 1PG, UK

## Abstract

Ubiquitylation is a covalent post-translational modification that regulates protein stability and is involved in many biological functions. Proteins may be modified with mono-ubiquitin or ubiquitin chains. Viruses have evolved multiple mechanisms to perturb the cell ubiquitin system and manipulate it to their own benefit. Here, we report ubiquitylation of vaccinia virus (VACV) protein N1. N1 is an inhibitor of the nuclear factor NF-κB and apoptosis that contributes to virulence, has a Bcl-2-like fold, and is highly conserved amongst orthopoxviruses. The interaction between N1 and ubiquitin occurs at endogenous protein levels during VACV infection and following ectopic expression of N1. Biochemical analysis demonstrated that N1 is covalently ubiquitylated, and heterodimers of ubiquitylated and non-ubiquitylated N1 monomers were identified, suggesting that ubiquitylation does not inhibit N1 dimerization. Studies with other VACV Bcl-2 proteins, such as C6 or B14, revealed that although these proteins also interact with ubiquitin, these interactions are non-covalent. Finally, mutagenesis of N1 showed that ubiquitylation occurs in a conventional lysine-dependent manner at multiple acceptor sites because only an N1 allele devoid of lysine residues remained unmodified. Taken together, we described a previously uncharacterized modification of the VACV protein N1 that provided a new layer of complexity to the biology of this virulence factor, and provided another example of the intricate interplay between poxviruses and the host ubiquitin system.

## Introduction

Ubiquitylation is a post-translational modification consisting of the covalent attachment of an ~8 kDa ubiquitin (Ub) protein onto a recipient protein. This process involves the sequential action of at least three cellular enzymes, E1, E2 and E3, the third of which provides the specificity to target the desired protein (Komander & Rape, 2012; Pickart, 2001). Ubiquitylation is a reversible process due to the action of deubiquitinases. Conjugation of Ub occurs via the coupling of the C-terminal glycine of Ub to internal lysine residues within the substrate, although other target residues (such as cysteines, threonines, serines and terminal amino groups) can also be ubiquitylated non-canonically. Ubiquitylation can occur at a single (mono-ubiquitylation) or multiple (multi-ubiquitylation) acceptor sites within the same target protein. In addition, the lysine residues of Ub can themselves be ubiquitylated, leading to the formation of Ub chains. These chains can contain from two to >10 Ubs that can have additional complexity due to the varying linkages between these molecules. Ub chains may mark proteins for proteasomal degradation, particularly those formed via the Lys48 of ubiquitin and, to a lesser extent, Lys11 (Komander & Rape, 2012; Pickart, 2001). However, an expanding body of evidence indicates that Ub chains also have essential roles in endocytosis, trafficking or signalling, amongst others (Bhoj & Chen, 2009; Gerlach *et al.*, 2011; Welchman *et al.*, 2005).

Several viruses have evolved mechanisms to manipulate the Ub system and to regulate it in an advantageous manner. They may encode Ub ligases and deubiquitinases, and hijack components of the Ub machinery to target cellular components (reviewed by Gustin *et al.*, 2011; Randow & Lehner, 2009). In addition, viruses can use ubiquitylation as a reversible post-translational modification to fulfil multiple biological functions with a limited number of proteins. For example, the human immunodeficiency virus protein Gag (Demirov *et al.*, 2002; Garrus *et al.*, 2001) and the Ebola virus protein VP40 (Yasuda *et al.*, 2003) are ubiquitylated for virion budding and release, and ubiquitylation and deubiquitylation of influenza NP protein correlates with viral replication (Liao *et al.*, 2010). Alternatively, Ub conjugation serves as a cellular mechanism to restrict pathogen propagation by degrading viral components as reported for the flavivirus NS5 protein (Taylor *et al.*, 2011) or the human papilloma virus protein E7 (Kamio *et al.*, 2004).

Poxviruses are large dsDNA viruses that replicate in the cytoplasm and express numerous proteins to manipulate the host cell environment (Moss, 2007). Poxviruses modulate the Ub system by multiple mechanisms (reviewed by Barry *et al.*, 2010; Shchelkunov, 2010; Zhang *et al.*, 2009): they code for their own Ub ligases such as p28 (Guerin *et al.*, 2002; Nerenberg *et al.*, 2005), express adaptors of cellular Ub complexes such as kelch and ankyrin proteins (Mohamed *et al.*, 2009; Sonnberg *et al.*, 2008; Sperling *et al.*, 2008; van Buuren *et al.*, 2008; Wilton *et al.*, 2008), or inhibit directly the function of cellular E3 ligases such as A49 or PACR (Mansur *et al.*, 2013; Mo *et al.*, 2009). In addition, research with vaccinia virus (VACV), the most intensively studied poxvirus and the vaccine employed to eradicate smallpox, has demonstrated the requirement for active proteasomes to complete the viral life cycle (Mercer *et al.*, 2012; Satheshkumar *et al.*, 2009; Teale *et al.*, 2009).

VACV encodes multiple proteins that promote immune evasion, called immunevasins (Smith *et al.*, 2013). One of them, N1, is a 14 kDa intracellular protein that is expressed early during infection and contributes to virulence (Bartlett *et al.*, 2002). Structurally, N1 is a homodimer and has a Bcl-2-like fold (Aoyagi *et al.*, 2007; Cooray *et al.*, 2007). Five other VACV proteins (B14, A52, F1, K7 and A46) have also been shown to have a Bcl-2 structure (Fedosyuk *et al.*, 2014; Graham *et al.*, 2008; Kalverda *et al.*, 2009; Kvansakul *et al.*, 2007) and other VACV proteins are predicted to be members of this family (González & Esteban, 2010; Graham *et al.*, 2008). VACV Bcl-2 proteins have diverse functions including inhibition of apoptosis, inflammasome activation and signalling cascades leading to activation of the nuclear factor NF-κB and IFN regulatory factor 3 (IRF-3) (reviewed by Smith *et al.*, 2013). Protein N1 was reported to inhibit the activation of IRF3 and NF-κB (DiPerna *et al.*, 2004), and although the inhibition of NF-κB has been confirmed (Chen *et al.*, 2008; Cooray *et al.*, 2007; DiPerna *et al.*, 2004; Maluquer de Motes *et al.*, 2011), the mechanism by which N1 mediates this inhibition is uncertain. N1 can also protect cells against apoptosis via a surface groove that is conserved in cellular anti-apoptotic proteins, but is occluded in VACV Bcl-2-like proteins that lack anti-apoptotic activity (Cooray *et al.*, 2007; Graham *et al.*, 2008; Maluquer de Motes *et al.*, 2011). The ability of N1 to prevent apoptosis has been disputed (Postigo & Way, 2012).

Here, we describe an additional feature of VACV protein N1. Whilst investigating potential cellular interacting partners for N1 using a recombinant VACV expressing an epitope-tagged N1, we noted that N1 exhibited multiple forms during infection and these represented ubiquitylated N1 proteins. N1 was ubiquitylated both during viral infection and following ectopic expression, and ubiquitylation was only abolished after mutagenesis of all N1 lysine residues. This previously uncharacterized interaction of N1 and ubiquitin added a new layer of complexity to the VACV protein N1, and provides another example of the interplay between poxviruses and the host Ub system.

## Results

### Generation of a recombinant tandem affinity purification (TAP)-tagged N1 virus

**Fig. 1.**
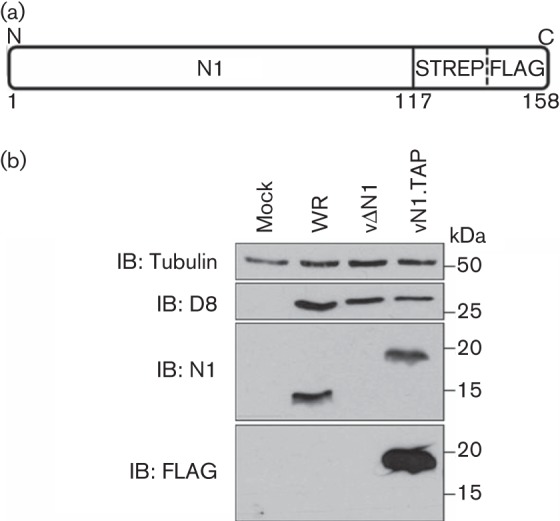
Generation of a recombinant TAP-tagged N1 virus. (a) Fusion of a TAP [streptavidin (STREP) and FLAG] tag at the C terminus of N1. (b) Infection of BS-C-1 cells with VACV strain WR, a recombinant VACV lacking the *N1L* gene (vΔN1) or a recombinant VACV expressing TAP-tagged N1 (vN1.TAP) at 2 p.f.u. per cell for 16 h. Whole-cell lysates were resolved by SDS-PAGE and immunoblotted (IB) with the indicated antibodies. Molecular mass markers are also included.

VACV protein N1 has multiple functions, some of which are not understood mechanistically. To gain better insight into how N1 performs these functions, a recombinant VACV strain, Western Reserve (WR), was generated by transient dominant selection (see Methods) in which the *N1L* gene was fused at the 3′ end to DNA encoding a TAP tag (vN1.TAP) consisting of a streptavidin-binding sequence and a FLAG epitope (Gloeckner *et al.*, 2007) (Fig. 1a). This virus expressed N1 at a similar level to WT VACV as revealed by immunoblotting using an anti-N1 rabbit serum (Bartlett *et al.*, 2002), indicating that the fusion did not affect the expression or stability of the protein (Fig. 1b). In addition, only protein N1 expressed from vN1.TAP infection could be detected by immunoblotting using an anti-FLAG antibody (Fig. 1b).

### N1 interacts with ubiquitin during viral infection

**Fig. 2.**
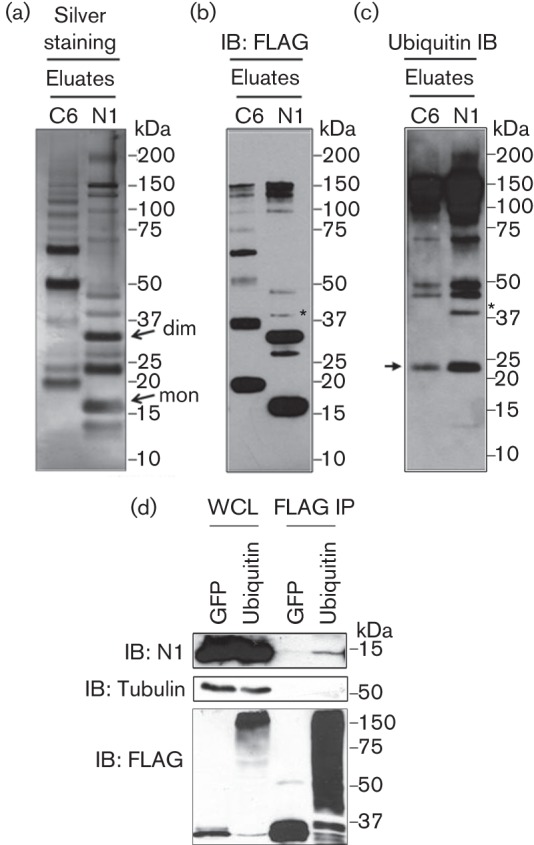
(a–c) N1 interacts with ubiquitin during infection. RAW247.1 macrophages were infected at 5 p.f.u. per cell with vC6.TAP and vN1.TAP, and subjected to TAP pull-down. Final eluates were resolved in Novex 4–12 % Bis-Tris protein gels and subjected to silver staining (a), or immunoblotting (IB) with anti-FLAG (b) or anti-ubiquitin (c) antibodies. A band of ~40 kDa that is recognized by anti-FLAG and anti-Ub antibodies is marked with an asterisk. The positions of the monomer (mon) and dimer (dim) are marked with arrows in (a). The position of the IgG light chain (LC) is indicated in (c). (d) Immunoprecipitation of FLAG-tagged Ub or GFP in cells infected with VACV WR. Whole-cell lysates (WCL) and FLAG immunoprecipitates (FLAG IP) were resolved by SDS-PAGE and immunoblotted with the indicated antibodies. Molecular mass markers are also included. Data shown are representative of at least two experiments showing similar results.

To identify N1 binding partners, RAW247.1 cells (murine macrophages) were infected with vN1.TAP or vC6.TAP, a control virus in which the C6 protein was tagged in the same way (Methods), at 2 p.f.u. per cell for 16 h. The cell lysates were subjected to sequential affinity purification, and concentrated protein eluates were fractionated in Novex 4–12 % Bis-Tris protein gels and analysed by silver staining or subjected to SDS-PAGE and immunoblotting. In the silver-stained gels, numerous bands were observed for both N1 and C6 that were unique for each protein (Fig. 2a). For N1, intense bands were observed at ~16 and 32 kDa, which were consistent with the expected size of monomeric and dimeric N1.TAP, respectively (Bartlett *et al.*, 2002; Maluquer de Motes *et al.*, 2011). Accordingly, these bands were detected by an anti-FLAG antibody (Fig. 2b). MS analysis further confirmed the identity of these bands as monomeric and dimeric N1 (data not shown).

FLAG immunoblotting also revealed other bands that did not correlate with predicted oligomers of N1. In particular, two bands were noted above the N1 dimer with sizes of ~40 and 48 kDa, and differing in ~8 kDa between each other and the N1 dimer. We speculated that these bands might correspond to ubiquitylated forms of the N1 dimer. To address this, samples were blotted with a mouse anti-Ub antibody and this showed the ~40 kDa band to be positive for Ub (Fig. 2c, asterisk). Unfortunately, we could not determine if the ~48 kDa band was ubiquitylated N1 due to non-specific signal present in both the N1 and C6 samples. These results revealed a previously unnoticed interaction between N1 and endogenous Ub in VACV-infected cells.

To confirm this interaction, a reciprocal immunoprecipitation was conducted. After FLAG immunoprecipitation, N1 co-immunoprecipitated with FLAG-Ub, but not FLAG-GFP (Fig. 2d), and this interaction was with monomeric N1 (the possible presence of ubiquitylated N1 forms could not be assessed due to non-specific signal when immunoblotting with anti-N1 serum). Therefore, N1 monomers may be immunoprecipitated with Ub due to their dimerization with ubiquitylated N1 monomers. In conclusion, N1 associated with Ub during viral infection.

### Interaction between N1 and Ub does not require virus infection

**Fig. 3.**
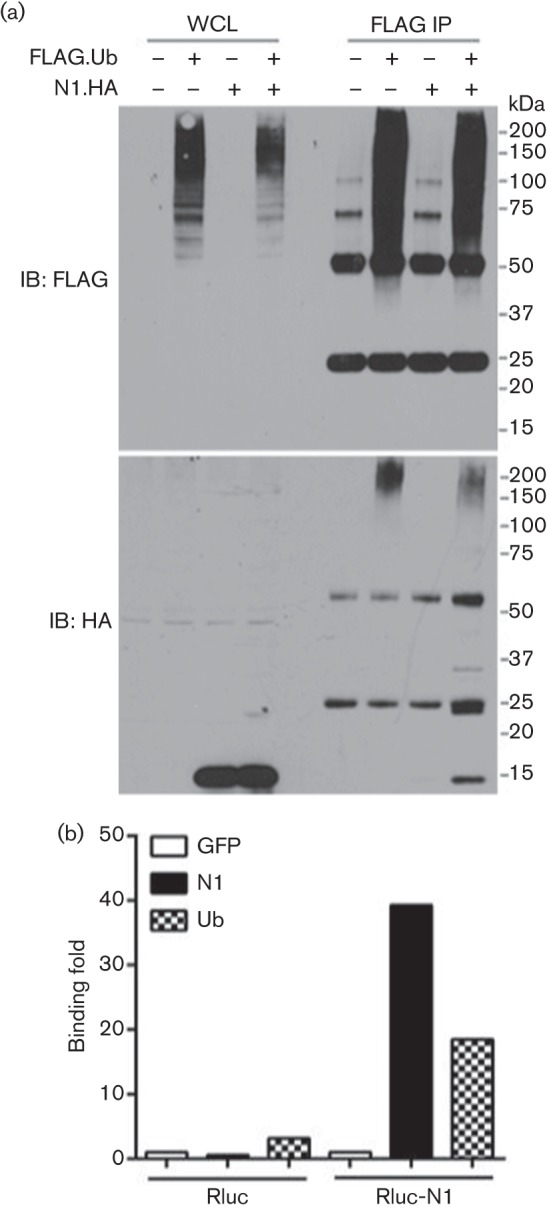
Interaction between N1 and Ub in transfected HEK 293T cells. (a) Immunoprecipitation of FLAG-tagged Ub in the presence (+) or absence (–) of HA-tagged N1. Whole-cell lysates (WCL) and FLAG immunoprecipitates (FLAG IP) were resolved in Novex 4–12 % Bis-Tris protein gels and immunoblotted (IB) with the indicated antibodies. The positions of molecular mass markers are indicated. (b) Interaction between FLAG-tagged versions of GFP, N1 or Ub and Rluc fused to N1 (Rluc-N1) or RLuc was assessed by LUMIER. Ratios between luciferase activity in lysates and eluates were calculated for each condition and plotted as a binding fold over control reactions. Data shown are from one experiment of three experiments showing indistinguishable results.

To determine whether the interaction between N1 and Ub was dependent on virus infection, HEK 293T cells were co-transfected with FLAG-tagged Ub and haemagglutinin (HA)-tagged N1 plasmids, and 24 h later cell lysates were subjected to FLAG immunoprecipitation followed by anti-HA immunoblotting. Up to three HA-positive bands were observed after co-transfection of N1 and Ub (Fig. 3a). Two of these bands matched the size of ubiquitylated N1 molecules (~23 and ~30 kDa).

Independently, the N1–Ub interaction was analysed by LUMIER (luminescence-based mammalian interactome mapping; Barrios-Rodiles *et al.*, 2005), taking advantage of a plasmid that codes for N1 fused with *Renilla* luciferase (Rluc) at its N terminus (Maluquer de Motes *et al.*, 2011). HEK 293T cells were transfected with Rluc-N1, or Rluc only, together with FLAG-tagged versions of GFP, N1 or Ub. Luciferase activity was measured before and after FLAG immunoprecipitation, and the ratios for each condition were plotted as binding fold over a control reaction. Interaction was observed between Rluc-N1 and FLAG-N1 due to the ability of N1 to dimerize, and between Rluc-N1 and FLAG-Ub (Fig. 3b). These interactions were not present in the Rluc control reactions, and this confirmed that N1 and Ub could interact after ectopic expression in cells in the absence of viral infection.

### N1 is covalently ubiquitylated

**Fig. 4.**
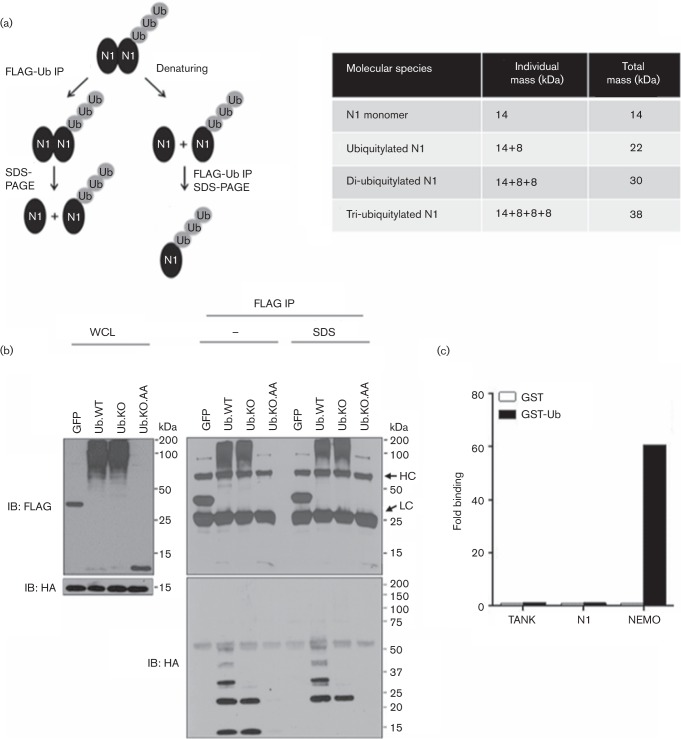
N1 is ubiquitylated. (a) Schematic of experimental design and the sizes of N1 and ubiquitylated derivatives. (b) HEK 293T cells were transfected with HA-tagged N1 and FLAG-tagged GFP or Ub.WT or Ub mutants, and lysates were subjected to immunoprecipitation under denaturing (SDS) or non-denaturing (–) conditions. Whole-cell lysates (WCL) and FLAG immunoprecipitates (FLAG IP) were resolved by SDS-PAGE and immunoblotted with the indicated antibodies. The HA immunoblot of the FLAG IP samples was resolved in Novex 4–12 % Bis-Tris protein gels. The positions of molecular mass markers and of the IgG light chain (LC) and heavy chain (HC) are indicated. (c) Interaction between Rluc-fused TANK, N1 or NEMO with recombinant GST-tagged Ub as assessed by LUMIER. Lysates from HEK 293T cells transfected with Rluc fusions were affinity purified with recombinant GST or GST-tagged Ub, and ratios between luciferase activity in lysates and eluates were calculated for each condition. Data were plotted as fold binding over the TANK control reaction. Data shown are from one experiment of three experiments showing indistinguishable results.

Ub is an ~8 kDa protein that is covalently attached and thus remains conjugated to its target after denaturation. To determine whether N1 was ubiquitylated, we performed an experiment in which lysates from HEK 293T cells transfected with HA-tagged N1 and FLAG-tagged Ub were boiled in the presence of 1 % SDS to destroy all non-covalent interactions, and subsequently immunoprecipitated and analysed by SDS-PAGE (illustrated in Fig. 4a). Two Ub mutants were also included: a mutant in which all lysine residues were replaced by arginines, but the two C-terminal glycines were left intact (Ub.KO), and a Ub.KO mutant in which the glycine residues were replaced by alanines (Ub.KO.AA). Given this mutagenesis, mutant Ub.KO could be attached to substrates and preformed Ub chains, but could not support Ub chain formation, whereas Ub.KO.AA would be impaired for both attachment and chain formation. Accordingly, after overexpression the input samples for Ub.WT and Ub.KO mostly resolved as a smear in the upper part of the gel, whereas Ub.KO.AA resolved only as a monomer (Fig. 4b, left). After FLAG immunoprecipitation of denatured lysates, an HA-positive band of ~22 kDa was detected for Ub.WT and Ub.KO, but not for Ub.KO.AA, indicating that this band was an N1 monomer covalently attached to a Ub molecule. Some higher-molecular-mass bands, separated by ~8 kDa, were also visible for Ub.WT, but not for Ub.KO, indicating that they were subsequent attachments of Ub moieties onto the first ubiquitylated N1 monomer. When the FLAG IP was performed under normal conditions, an additional HA-positive band appeared corresponding to monomeric non-ubiquitylated N1 (~14 kDa). This demonstrated that non-covalent interactions such as N1 dimerization were disrupted by the denaturing treatment applied and that non-ubiquitylated monomeric N1 could associate with ubiquitylated N1 molecules in cells. The latter also suggested that the co-precipitation of monomeric non-ubiquitylated N1 with ubiquitin observed in previous experiments (Fig. 2d) was mediated by ubiquitylated heterodimers.

To test whether N1 could also bind Ub non-covalently, we purified glutathione *S*-transferase (GST)-fused ubiquitin chains from bacteria and incubated them with lysates from cells expressing Rluc-N1. Cells expressing Rluc fusions for other unrelated cellular proteins (TANK and NEMO) were also included as negative and positive controls for the assay, respectively (Thurston *et al.*, 2009). Ratios of luciferase activity were measured before and after the GST pull-down and, as expected, these revealed the Ub-binding capacity of NEMO (Fig. 4c). However, no such ability was observed for N1. Taken together, these results indicate that the interaction between N1 and Ub is mediated by ubiquitylation rather than an ability to bind Ub.

### N1 does not accumulate after proteasome inhibition

**Fig. 5.**
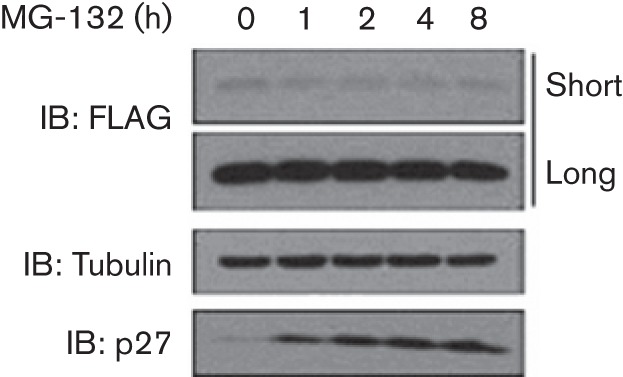
N1 is not targeted for proteasomal degradation. Lysates from HEK 293T cells transfected with TAP-tagged N1 and treated with MG-132 for the indicated lengths of time were resolved by SDS-PAGE and immunoblotted with the indicated antibodies. Short and long exposures are shown for the FLAG (N1) panel. Data shown are from one experiment of three experiments showing indistinguishable results.

Protein ubiquitylation is a crucial regulator of protein degradation in cells and most ubiquitylated proteins are targeted for degradation. Previous experiments had revealed that the levels of N1 protein were not altered by overexpression of Ub, suggesting that N1 ubiquitylation did not affect N1 stability or trigger its degradation. To confirm this, cells expressing N1 were treated with MG-132, an inhibitor of the proteasome, for different lengths of time and levels of N1 protein were determined by immunoblotting. No difference in N1 levels was observed after inhibition of the proteasome for up to 8 h (Fig. 5), suggesting that ubiquitylation of N1 did not trigger its proteasomal degradation. Conversely, p27, a well-known target of Skp2 that is constitutively degraded by the proteasome, accumulated progressively.

### Vaccinia proteins C6 and B14 are not ubiquitylated

**Fig. 6.**
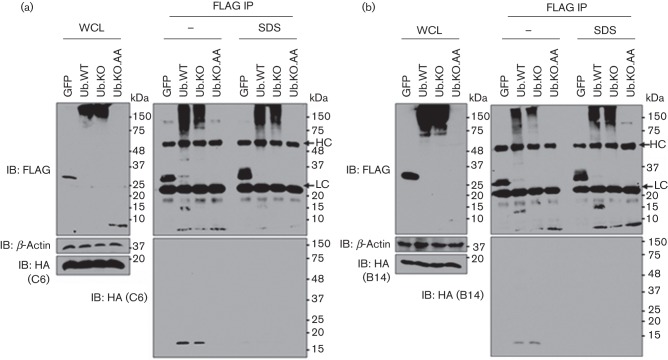
VACV proteins C6 and B14 are not ubiquitylated. (a, b) Immunoprecipitation of FLAG-tagged WT or mutant Ub after infection with recombinant VACVs expressing HA-tagged C6 (a) or B14 (b) under denaturing (SDS) and non-denaturing (–) conditions. Ub mutants lacking all lysines (Ub.KO) or lacking all lysines and the C-terminal glycines (Ub.KO.AA) were used. Whole-cell lysates (WCL) and FLAG immunoprecipitates (FLAG IP) were resolved by SDS-PAGE and immunoblotted with the indicated antibodies. The positions of the molecular mass marker and of the IgG light (LC) and heavy chain (HC) are indicated. Data shown are from one experiment of three experiments showing indistinguishable results.

To determine whether ubiquitylation was unique for N1 or could also occur on other members of the VACV Bcl-2-like family, proteins C6 and B14 were studied. Similarly, in previous experiments, cells expressing different FLAG-tagged Ub alleles were infected with recombinant VACVs expressing HA-tagged C6 (Unterholzner *et al.*, 2011) or HA-tagged B14 (Chen *et al.*, 2006). As expected, each Ub mutant migrated differently according to their properties (Fig. 6a). Both C6 (Fig. 6a) and B14 (Fig. 6b) immunoprecipitated with Ub under normal but not denaturing conditions, indicating that their interactions with Ub are non-covalent.

### N1 is ubiquitylated in a lysine-dependent manner

**Fig. 7.**
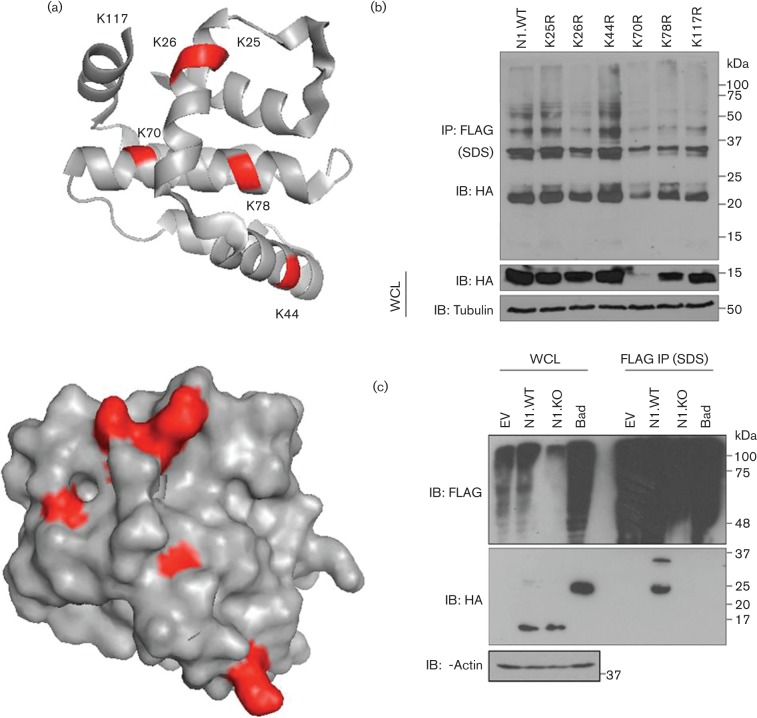
Ubiquitylation of N1 lysine mutants. (a) Structural depiction of the N1 monomer in a ribbon-based or surface drawing obtained using PyMOL software. Lysine residues are numbered and highlighted in red. Residue K117 was not resolved in the crystal structure (Protein Data Bank ID: 2I39). (b, c) Immunoprecipitation under denaturing conditions of FLAG-tagged WT Ub in the presence of HA-tagged N1 mutants. Single lysine N1 mutants (b) or lysine-free N1 (N1.KO) (c) were used and compared with WT N1. HA-tagged Bad was also included. Whole-cell lysates (WCL) and FLAG immunoprecipitates were resolved by SDS-PAGE and immunoblotted (IB) with the indicated antibodies. The positions of molecular mass markers are indicated. Data shown are from one experiment of two experiments showing indistinguishable results.

To further understand the role of N1 ubiquitylation, single lysine N1 mutants were generated and their ability to be ubiquitylated was studied. N1 contains six lysine residues at positions 25, 26, 44, 70, 78 and 117. All of these are located at the surface on the molecule (although residues 70 and 78 are exposed to a lesser degree) and therefore have the potential to be accessible to the ubiquitylating machinery (Fig. 7a). We mutated each lysine residue to arginine individually and overexpressed each mutant in cells together with FLAG-Ub. All mutants expressed to comparable levels as the WT with the exception of mutant K70R. After immunoprecipitation under denaturing conditions all mutants, including K70R, co-precipitated with Ub, indicating that they were all ubiquitylated (Fig. 7b). To test whether K25 and K26 could compensate for each other due to their proximity, a double mutant K25,26R was also tested and similar ubiquitylation to N1.WT was observed (data not shown). To assess whether N1 could have multiple lysine acceptor sites or whether ubiquitylation could occur at alternative residues other than lysines, a lysine-free N1 (N1.KO) construct was purchased. N1.KO was expressed at similar levels to N1.WT, but failed to be ubiquitylated after co-expression with FLAG-tagged Ub and immunoprecipitation under denaturing conditions (Fig. 7c). In addition, protein Bad, a cellular protein with a Bcl-2-like fold, was included in the assay and failed to be ubiquitylated under the conditions tested, indicating that ubiquitylation was specific to N1 rather than the Bcl-2-like fold. Collectively, these data showed that N1 is covalently linked to Ub via one or more lysine residues.

**Fig. 8.**
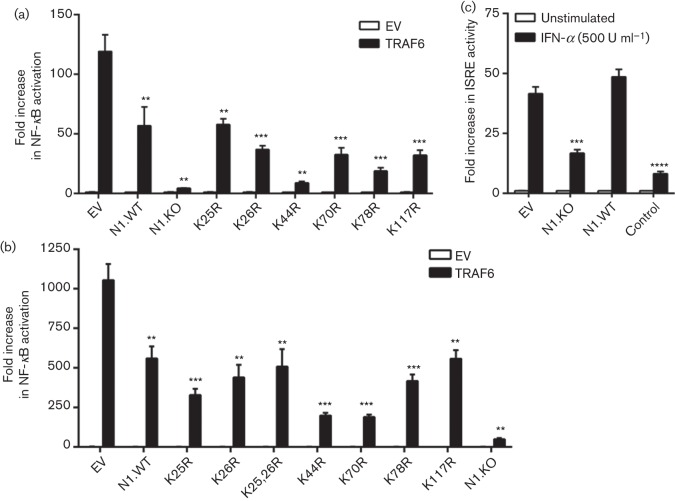
Inhibition of NF-κB and ISRE reporter activity by N1 lysine mutants. (a, b) Cells were transfected with the indicated plasmids together with pNF-κB-Luc and pTK-Ren. NF-κB activity was triggered by co-transfection with TRAF6. Cells were harvested 24 (a) or 36 h (b) post-transfection, and luciferase activity was plotted as fold activation over control conditions. (c) Cells were transfected with the indicated plasmids together with pISRE-Luc and pTK-Ren, and stimulated with IFN-α (500 U ml^−1^) for 8 h. Data are mean±sd with statistical analysis (Student’s *t*-test, **P<0.01, ***P<0.005, ****P<0.001) and are representative of at least two experimental repeats each. EV, Empty vector.

To determine whether ubiquitylation of N1 could affect its function, N1 lysine mutants were transfected in HEK 293T together with a reporter expressing luciferase under control of the NF-κB consensus promoter sequence. NF-κB activation was triggered by co-transfection with TNF-associated factor 6 (TRAF6) and luciferase activity was measured after 24 h (Fig. 8a) or 36 h (Fig. 8b). In both assays, all N1 lysine mutants retained the ability to inhibit NF-κB activation. However, N1.KO showed the greatest inhibition. To characterize this mutant further, N1.KO was tested in reporter assays where N1 was known not to have inhibitory activity. HEK 293T cells were transfected with a reporter containing IFN-stimulated reporter element (ISRE) together with N1.WT, N1.KO or a control molecule. Unexpectedly, N1.KO blocked ISRE activity upon treatment with IFN-α. These data demonstrated that N1.KO inhibited non-specifically the activation of unrelated reporter assays for reasons that remain unknown.

## Discussion

In this study, a VACV expressing TAP-tagged N1 was generated to search for N1-interacting partners by TAP pull-down coupled to MS. Although no binding partners whose activity could be linked to the biological activities reported for N1 were found, Ub was specifically identified as an N1-interacting partner by immunoblotting (Fig. 2). To confirm this interaction, conventional immunoprecipitation was performed and this demonstrated that the interaction occurred not only with endogenous Ub during viral infection, but also after ectopic expression in transfected cells (Fig. 3). Furthermore, biochemical experiments revealed that N1 is directly and covalently ubiquitylated (Fig. 4b), and does not bind purified recombinant Ub (Fig. 4c), which is consistent with the lack of Ub-binding domains in the Bcl-2 structural fold (Chipuk *et al.*, 2010). Given this, the non-covalent interactions observed between Ub and VACV proteins B14 and C6 are likely to be indirect and mediated by a third cellular or viral protein(s).

Ubiquitylation can occur in multiple forms: conjugation of a single Ub monomer (mono-ubiquitylation), conjugation of multiple monomers at different acceptor sites (multi-mono-ubiquitylation), conjugation of subsequent monomers onto each other to form Ub chains and combinations of these (Komander, 2009). In our experiments, Ub laddering was observed after denaturing. This could be caused by the conjugation of ubiquitin chains or by the simultaneous presence of multiple Ub monomers at different sites. Using a lysine-free Ub molecule that could not support chain elongation, we determined the formation of Ub chains, rather than multi-mono-ubiquitylation, on N1 (Fig. 4b). Ubiquitylation is commonly known as a cellular mechanism to target proteins for degradation. This is normally achieved by the formation of Lys48 Ub chains (Pickart, 2001). Although Ub chains were observed for N1, our attempts to determine the linkage between the different Ub monomers were inconclusive (data not shown). To investigate if N1 was targeted for proteasomal degradation, pharmacological inhibitors of the proteasome were used and such treatment did not affect the levels of N1 protein (Fig. 5).

Poxvirus protein N1 contains six lysine residues, and the crystal structure of N1 (Aoyagi *et al.*, 2007; Cooray *et al.*, 2007) showed that all of these are near the protein surface. Lys25, 26, 44 and 117 are very exposed, whereas Lys70 and 78 are in partially hidden positions (Fig. 7a), and the mutation of K70R reduced protein stability. Mutagenesis studies were performed to pinpoint which lysine residue was responsible for N1 ubiquitylation. These demonstrated that ubiquitylation on N1 could not be abrogated after mutagenesis of any single lysine residue, suggesting that more than one lysine can act as a lysine recipient (Fig. 7). However, replacement of all lysines prevented ubiquitylation, indicating that ubiquitylation of N1 occurs in a conventional lysine-dependent manner, with no involvement of non-canonical residues including the N-terminal group. Also, this suggests that even if a preferential acceptor site exists, alternative lysine residues can be used for Ub attachment. Identification of these residues and of their relative importance will require further studies.

Poxvirus protein N1 has been reported to contain at least two different functions: it can prevent apoptosis (Cooray *et al.*, 2007; Graham *et al.*, 2008; Maluquer de Motes *et al.*, 2011) and it can inhibit intracellular signalling, particularly activation of NF-κB (Chen *et al.*, 2008; Cooray *et al.*, 2007; DiPerna *et al.*, 2004; Maluquer de Motes *et al.*, 2011). Inhibition of apoptosis by N1 has recently been mapped to a surface groove that mimics those of cellular anti-apoptotic Bcl-2 proteins (Maluquer de Motes *et al.*, 2011). Mutations that limit the accessibility to this groove prevent N1 from binding cellular pro-apoptotic proteins Bad and Bid, and consequently from preventing cell death. In contrast, the mechanism employed by N1 to interfere with intracellular signalling remains unclear. Given this and the importance of Ub in the regulation of inflammatory signalling (Bhoj & Chen, 2009; Gerlach *et al.*, 2011), it is tempting to speculate a correlation between the ubiquitylation of N1 and its ability to inhibit NF-κB. Unfortunately, functional analysis using lysine-free N1 revealed non-specific, off-target inhibition of different unrelated reporter activities for unknown reasons. Thus, whether there is a formal relationship between N1 ubiquitylation and the inhibition of inflammatory signalling remains unknown. Nonetheless, previous work reported that mutation I6E in N1 abrogated dimer formation and the ability to inhibit NF-κB by N1 (Maluquer de Motes *et al.*, 2011). This indicated that either N1 needs to be a dimer to block NF-κB or that the same interface in N1 is used for both functions. No lysines are present in this interface, suggesting that ubiquitylation of N1 might not interfere with dimer formation. Given that ubiquitylation can occur after dimerization, further experiments are required to determine the ability of ubiquitylated N1 monomers to dimerize *de novo*.

Identification and characterization of viral proteins that undergo ubiquitylation is important to understand the complex mechanisms employed by viruses to modulate the host cell environment. Ub and Ub-like molecules are involved in many aspects of cell biology, including endocytosis, trafficking, transcription and the innate immune response. Given that viruses manipulate host cell machinery, interplay between viruses and the Ub system is to be expected. For some aspects, such as retrovirus budding and egress (Martin-Serrano, 2007) or degradation of tumour suppressor protein p53 (Beaudenon & Huibregtse, 2008; Mammas *et al.*, 2008), it is clear that viruses have advantageously hijacked the cellular apparatus to facilitate the viral life cycle. For some others, however, it is difficult to distinguish between viral manipulation of host machinery and cellular inhibition of viral replication, or even adventitious modification of viral proteins. In this work, we demonstrate that VACV virulence factor N1 is ubiquitylated. Similar ubiquitylation has also been demonstrated for VACV protein E3 (González-Santamaria *et al.*, 2011). Interestingly, both N1 and E3 are intracellular factors with multiple functions. Post-translational ubiquitylation may thus account for the diverse biological effects mediated by these proteins during VACV infection.

## Methods

### 

#### Cells, viruses and reagents.

BS-C-1, HEK 293T and RAW247.1 macrophages were grown at 37 °C in a 5 % CO_2_ atmosphere in Dulbecco’s modified Eagle’s medium supplemented with 10 % FBS (Invitrogen), 100 U penicillin ml^−1^ (Invitrogen) and 100 mg streptomycin ml^−1^ (Invitrogen). vC6.HA and vB14.HA were as described previously (Chen *et al.*, 2006; Unterholzner *et al.*, 2011). MG-132 (Sigma-Aldrich) was dissolved in DMSO and used at 20 µM. IFN-α was from Peprotech.

#### Expression plasmids.

HA-tagged N1 and Rluc -fused N1 were as described previously (Maluquer de Motes *et al.*, 2011). Site-directed mutagenesis of both the HA-tagged N1 and FLAG-tagged Ub was performed using the QuikChange mutagenesis kit (Stratagene). The N1 allele where all lysine residues had been replaced by arginines (N1.KO) was obtained from Geneart (Invitrogen). FLAG-tagged GFP, TRAF6 and Rluc fusions for TANK and NEMO were gifts from F. Randow (MRC Laboratory of Molecular Biology, UK). HA-tagged Bad was from Xin Lu (Ludwig Institute for Cancer Research, UK). FLAG-tagged Ub was from Christel Brou (Institute Pasteur, France). All sequences were verified by DNA sequencing.

#### Generation of vN1.TAP.

N1 fused with a C-terminal TAP tag (Maluquer de Motes *et al.*, 2011), as well as the flanking regions of the *N1L* gene of the VACV WR strain, were cloned into the transfer vector pUC13 containing EGFP and EcoGPT selection/marker genes as described previously (Ember *et al.*, 2012). BS-C-1 cells infected with WR lacking N1 (vΔN1) (Bartlett *et al.*, 2002) were transfected with pUC13-N1.TAP using Fugene (Roche) and recombinant viruses were selected as described previously (Maluquer de Motes *et al.*, 2011; Unterholzner *et al.*, 2011). Recombinant vN1.TAP viruses were screened by PCR using primers located in the flanking regions, and finally expanded and titrated by plaque assay in BS-C-1 cells. Protein expression was analysed after infection of BS-C-1 cells for 16 h with 2 p.f.u. per cell. A recombinant virus expressing TAP-tagged C6 was generated in a similar manner after cloning of the *C6L* gene and its flanking regions into the pUC13 vector.

#### TAP.

TAP was performed as described previously, with minor modifications (Gloeckner *et al.*, 2007; Peters *et al.*, 2013). RAW247.1 macrophages were infected overnight with 2 p.f.u. per cell and subsequently lysed in PBS supplemented with 0.5 % NP-40. Lysates were incubated with streptavidin beads (Thermo Scientific) for 2 h at 4 °C. Beads were washed three times and eluted in PBS supplemented with biotin (Thermo Scientific) for 1 h at 4 °C. Eluates were incubated with anti-FLAG M2 agarose beads (Sigma) and eluted in PBS supplemented with FLAG peptide (Sigma) in a similar manner. Final eluates were concentrated using 3 kDa Amicon filters (Millipore). Proteins retained on streptavidin and FLAG beads, as well as final eluates, were resolved in Novex 4–12 % Bis-Tris protein gels (Invitrogen) and analysed by silver staining (Invitrogen), or immunoblotting with anti-FLAG (Sigma-Aldrich) or anti-ubiquitin (FK2; Enzo Life Sciences) antibodies.

#### Immunoprecipitation and LUMIER.

HEK 293T cells were transfected using calcium chloride and lysed 24 h later with IP buffer (20 mM Tris/HCl, pH 7.4, 150 mM NaCl, 10 % glycerol, 0.1 % Triton X-100 and protease inhibitors; Roche) as described previously (Maluquer de Motes *et al.*, 2011). Whole-cell lysates and immunoprecipitates were analysed in Novex 4–12 % Bis-Tris protein gels (Invitrogen) or by home-made SDS-PAGE and immunoblotting with the following antibodies: anti-N1 (Bartlett *et al.*, 2002), anti-D8 (Parkinson & Smith, 1994), anti-FLAG (Sigma-Aldrich), anti-tubulin (Upstate Biotech), anti-HA (Covance), anti-p27 (Cell Signalling) and anti-β-actin (Abcam). For LUMIER with GST fusions, GST and GST-fused Ub were expressed in BL21 bacterial cells, lysed in BugBuster lysis buffer (Novagen), and purified by affinity chromatography as described previously (Thurston *et al.*, 2009). Recombinant protein (50 ng per sample) was incubated with GST Sepharose beads (Amersham Pharmacia) for 1 h at 4 °C and mixed with cell lysates containing Rluc fusions. Samples were further incubated for 2 h at 4 °C and washed three times with lysis buffer. GST complexes were eluted in Passive Lysis Buffer (Promega) supplemented with 100 mM glutathione (Amersham Pharmacia) and luciferase activity was measured. Ratios between the whole-cell lysates and the GST pull-down eluates were calculated and compared with controls.

#### Ubiquitylation assay.

To examine ubiquitylation, HEK 293T cells were transfected using calcium chloride and/or infected with the indicated viruses, and 24 h later were lysed in TNE buffer (50 mM Tris/HCl, pH 7.5, 250 mM NaCl, 1 % NP-40, 1 mM EDTA and 1 mM DTT) supplemented with *N*-ethylmaleimide (Sigma-Aldrich) as described previously (Yamazaki *et al.*, 2009). Cell lysates were centrifuged (10 000 ***g*** for 30 min) and cleared supernatants were mixed with 1 vol. 2 % SDS TNE. Samples were heated at 90 °C for 10 min to destroy all non-covalent interactions. Lysates were diluted 10-fold in TNE buffer and subjected to FLAG immunoprecipitation for 16 h using FLAG M2 resin (Sigma-Aldrich). Samples were washed three times in TNE buffer and finally analysed by immunoblotting.

#### Reporter gene assays.

HEK 293T cells were transfected with 100 ng per well of the indicated plasmids together with 70 ng per well of pNF-κB-Luc and 10 ng per well of pTK-Ren using Fugene (Roche). NF-κB was triggered by co-transfection of 10 ng per well of TRAF6. Luciferase activity was measured 24 or 36 h later and expressed as fold activation over controls. Similarly, HEK 293T cells were transfected with the indicated plasmids together with pISRE-Luc and stimulated with IFN-α for 8 h.
